# Ghrelin, Appetite Regulation, and Food Reward: Interaction with Chronic Stress

**DOI:** 10.1155/2011/898450

**Published:** 2011-09-21

**Authors:** Yolanda Diz-Chaves

**Affiliations:** Instituto Cajal, CSIC, Avenida Doctor Arce 37, 28002 Madrid, Spain

## Abstract

Obesity has become one of the leading causes of illness and mortality in the developed world. Preclinical and clinical data provide compelling evidence for ghrelin as a relevant regulator of appetite, food intake, and energy homeostasis. In addition, ghrelin has recently emerged as one of the major contributing factors to reward-driven feeding that can override the state of satiation. The corticotropin-releasing-factor system is also directly implicated in the regulation of energy balance and may participate in the pathophysiology of obesity and eating disorders. This paper focuses on the role of ghrelin in the regulation of appetite, on its possible role as a hedonic signal involved in food reward, and on its interaction with the corticotropin-releasing-factor system and chronic stress.

## 1. Introduction

Obesity and related disorders are among the leading causes of illness and mortality in the developed world, and it became a severe epidemic problem not only in adults but also in younger generations [1]. To better understand the pathophysiological mechanism that underlies metabolic disorders, increasing attention has been paid to central regulatory elements in energy homeostasis, including food intake and energy expenditure. The discovery of ghrelin and its influence on appetite, fuel utilization, body weight, and body composition adds yet another component to the complexity in the central regulation of energy balance [2]. In addition, recently it has been described that ghrelin may be implicated in food reward [3], since ghrelin directly targets the ventral tegmental area to increase food motivation [4]. However, it remains to be determined whether the central ghrelin signaling system has a role in the pathophysiology of obesity.

The corticotropin releasing factor (CRF) system is directly implicated in the regulation of energy balance [5, 6]. In addition, it may participate in the pathophysiology of obesity and eating disorders [7, 8]. Considerable evidence has accumulated to demonstrate that feeding behavior is influenced by stress. Stress triggers a physiological response that involves the coordinated control of multiple motor, hormonal, and vegetative systems that are engaged to reestablish homeostasis in the face of the stress perturbation, either real or perceived, of the internal and/or external environment [9]. In human beings, many obese patients tend to eat more when they are emotionally tense or depressed or simply bored. Sadness favored eating of high fat/sweet, hedonically rewarding foods, whereas intake during a happy state favored dried fruit [1]. In addition, it has been described that visceral overweight subjects showed stress-induced food intake in the absence of hunger, resulting in an increased energy intake [10].

Increasing evidence suggests an interplay between the CRF and ghrelin systems. However, it is unknown wether this interacion plays a role in the pathophysiology of obesity.

## 2. Ghrelin and the Regulation of Energy Balance

Small synthetic molecules called growth hormone secretagogues (GHSs) stimulate the release of growth hormone (GH) from the pituitary. They act through the GHS-receptor (GHS-R), which has been cloned for different groups [11, 12]. Despite intensive searches by different groups, in 1999, was purified and identified the endogenous specific ligand for GHS-R [13]. This ligand is a molecule of 28 amino acids called ghrelin in which the serine residue 3 was n-octanoylated. The minimum core of ghrelin residing in the N-terminal tetrapeptide and the acylation of the peptide had been supposed critical to cross the blood-brain barrier but is also essential for binding the GHS-1a receptor and for its GH releasing and other endocrine actions [13–15]. Recently, the enzyme responsible for the acylation of ghrelin was identified by two independent groups [16, 17]. The ghrelin O-acetyltransferase (GOAT) is the specific acyl transferase that activates ghrelin [16, 17] and belongs to the super family of membrane-bound O-acyltransferases (MBOATs) [17–19]. In humans, GOAT expression is high in stomach and gut, the major ghrelin-secreting tissues, and in the pituitary, in which ghrelin is known to show autocrine and paracrine effects [16, 20]. GOAT is regulated by nutrient availability, depends on specific dietary lipids as acylation substrates and links ingested lipids to energy expenditure and body fat mass [21]. Although devoid of neuroendocrine activity, also unacylated ghrelin, its most abundant circulating form, is an active molecule. This evidence agrees with the existence of GHS-R subtypes that are activated by ghrelin independently of its acylation [13, 22]. 

In humans, the stomach is a major source of circulating ghrelin. It has been shown that plasma ghrelin-like immunoreactivity levels in gastrectomized patients still remain 35% of those in normal subjects, suggesting that tissues other than the stomach contribute to a certain amount of circulating ghrelin [23]. The peptide is produced prevalently in the stomach by the X/A-like cells within the oxyntic glands of the gastric fundus mucosa [13, 24–27], with substantially lower amounts derived from the bowel [24, 27–29], pancreas [28, 30], lymphocytes [28], chondrocytes [31], kidney [28, 32], placenta [33], lung [28], testis [28, 34], ovary [35, 36], adrenal cortex [37, 38], pituitary (lactotrophs, somatotrophs, and thyrotrophs cells) [39, 40], and hypothalamus [40–42].

Ghrelin is strongly involved in the regulation of energy homeostasis. The group of Ghigo and coworkers published for the first time the involvement of ghrelin in the regulation of appetite. They described that 3 out of 4 healthy volunteers reported hunger following ghrelin administration as a “side effect” in a clinical study analyzing GH release, and this effect has been confirmed in more studies [43, 44]. In animal studies, it has been shown that ghrelin increased food intake of rats, when it was administered either into central nervous system or peripherally [45–47], in both satiated and feeding conditions [48]. The i.c.v. administration generated a dose-dependent increase in food intake and body weight [49–51]. The time course and magnitude of this effect was similar to neuropeptide Y (NPY) [46, 49]. Also, it has been shown a significant increase of the food intake after the systemic administration of ghrelin, occurring at plasma ghrelin levels within normal fasting range [47, 52]. In rodents, ghrelin-induced gain weight is based on accretion of fat mass gain by reducing fat utilization, without changes in longitudinal skeletal growth and without an increase in lean [51]. 

Very low doses (30 pmol) of ghrelin injected into the arcuate nucleus (ARC) potently stimulated food intake. Another hypothalamic nucleus that showed a similarly prompt significant orexigenic response was the paraventricular nucleus (PVN), but the stimulation of food intake was significantly less than the one seen in the ARC [47]. Studies with confocal laser microscopy have analyzed the effect of ghrelin on the expression of c-fos, a marker of neuronal activation. These studies have shown that i.p. administration of ghrelin induces c-fos immunoreactivity in (i) the ARC and medioparvocellular part of the PVN [52, 53], (ii) the dorsomedial and lateral hypothalamic nuclei, and (iii) two regions of the brainstem, the nucleus of the tractus solitarius and the area postrema [53]. In addition, after i.c.v. ghrelin administration, c-fos immunoreactivity was found in neuronal populations of primary importance in the regulation of feeding, including NPY neurons, AgRP neurons [42, 48], and orexin-containing neurons (in the lateral hypothalamus) [53]. Furthermore, antibodies and antagonist of NPY and AgRP abolish ghrelin-induced feeding [54]. Ghrelin increases NPY and AgRP mRNA expression levels [42, 48–50, 55] independently of the nutritional status [56] and blocked leptin-induced feeding reduction [48]. Furthermore, the orexigenic effect of ghrelin is abolished by i.c.v. coinjection of Y1 receptor antagonist, indicating that ghrelin increases food intake at least partly through the activation of the hypothalamic NPY/Y1 pathway [50, 55]. 

Endogenous ghrelin secretion is pulsatile and directly related to feeding behavior [57]; after fasting, ghrelin secretion is augmented in the form of high frequency, high amplitude episodes [58, 59]. Ghrelin concentrations in the blood and mRNA levels were increased by fasting and decreased by feeding in the stomach [23, 51, 60]. The plasma levels of ghrelin inversely correlate with body mass index (BMI), thus, ghrelin levels are modified in patients with anorexia nervosa and obesity. Ghrelin secretion is increased in anorexia and cachexia, reduced in obesity, and normalized by recovery of ideal body weight [23, 61, 62]. The paradoxical situation of increased basal plasma ghrelin concentrations observed in subjects with long-term energy deficit and decreased food intake such as in restrictive anorexia nervosa [63] suggests the existence of ghrelin resistance that may be relevant to the decreased effects of ghrelin to promote a positive energy balance [64]. In addition, plasma ghrelin levels regulation could be influenced by ghrelin reactive autoantibodies (autoAbs), since it had been demonstrated the presence of autoAbs reactive with ghrelin in healthy subjects and rats [65, 66]. Furthermore, a role of autoAbs against appetite-regulating neuropeptides in eating disorders has emerged [65]. In this regard, it had been described decreased plasma levels of acyl ghrelin IgG autoAbs present as free or total immunoglobulins in patients with anorexia nervosa, suggesting that altered production of ghrelin reactive autoAbs is associated with persistently elevated plasma ghrelin and eventually ghrelin resistance in anorexia nervosa [67].

Furthermore, a negative association between body mass and ghrelin secretion in Prader-Willi syndrome exists, where obesity is associated with ghrelin hypersecretion [68]. In obese humans, food intake (including 60% of carbohydrates) with postprandially increased insulin response fails to suppress ghrelin levels sufficiently [69], suggesting that ghrelin may be involved in some of the multiple pathophysiological mechanisms leading to obesity and type 2 diabetes [70].

The reduction in plasma ghrelin levels with high-fat diet agreed with the low circulating levels of ghrelin in obese humans and db/db reflected a physiological adaptation to the positive energy balance associated with obesity [23, 62, 71, 72]. In addition, high-fat diet causes ghrelin resistance by reducing NPY/agouti-related peptide (AgRP) responsiveness to plasma ghrelin and suppressing the neuroendocrine ghrelin axis to limit further food intake [73]. Indeed, ghrelin secretion increases with a low-protein diet and decreases with a high-fat diet [26]. It has been shown that sugar intake, but not stomach expansion, reduced serum ghrelin levels [51].

It is clear that several molecular mediators are involved in the control of energy homeostasis. This is a highly complex process that involves several brain regions, ranging from cortex to brainstem. However, an important effort has been paid to understand the role of hypothalamus on this process, since numerous neural circuits involved in the homeostatic control are located in this brain region. Discrete neuronal populations in the hypothalamus are regulated by specific signals of nutritional state and express neurotransmitters that mediate particular effects on food intake and/or energy expenditure. These neuronal populations were considered to mediate “feeding” or “satiety” responses [74]. Currently, it seems likely that the effects that are causing a positive energy balance are mediated via leptin-responsive neurons in specific regions of the hypothalamus [2, 48, 50]. By in situ hybridization procedure, it has been shown that the effect of food intake on NPY neurons is mediated by the direct action of leptin via Ob-Rb receptors expressed by these NPY cells. The expression of these receptors is a defining phenotypic characteristic of the subset of NPY arcuate neurons that are activated by fasting and play an adaptive response to negative energy balance [75]. Leptin reduces food intake, body weight, and hypothalamic NPY mRNA expression, and these effects are reversed by the simultaneous administration of ghrelin through the activation of hypothalamic NPY/Y1 receptor pathway [48, 50, 55, 60]. However, although the leptin-induced decrease in hypothalamic NPY mRNA expression is completely abolished by i.c.v. coinjection of ghrelin, the satiety effect of leptin is only partially reversed by ghrelin. This suggests the involvement of other orexigenic or anorexigenic systems in the antagonism of leptin action by ghrelin [50].

Information of both anorexigenic systems, mainly including melanocortin-derived peptides and orexigenic peptidergic systems containing NPY and AgRP, seem to converge in PVN [76]. Ghrelin injected peripherally induces c-fos expression in arcuate NPY-positive neurons that project to the medioparvocellular part of PVN [77]. Cowley et al. showed that ghrelin is expressed in a previously uncharacterized group of neurons in the hypothalamus. These neurons lie in the space between the lateral, arcuate, ventromedial, dorsomedial, and PVN hypothalamic nuclei and they send projections to several of these nuclei as well as outside of the hypothalamus [41]. Ghrelin boutons established synaptic contacts on cell bodies and dendrites of NPY/AgRP and Proopiomelanocortin (POMC) neurons in the arcuate nucleus. Also, ghrelin boutons were in direct apposition to NPY and GABA axon terminals in the ARC and PVH. In the paraventricular hypothalamic nucleus (PVN), some of ghrelin axons innervated CRF cells [70, 78, 79]. Therefore, the final effects of ghrelin are likely to reflect complex interactions of GHRH, CRF, AVP, NPY, and other hypothalamic neuronal circuits [80].

In addition, it has been recently described that ghrelin is implicated in certain rewarding aspects of eating that are separated from eating-associated body weight homeostasis and that require the presence of intact orexin signaling [81]. Growth hormone secretagogue receptors are expressed in tegmental and mesolimbic areas, such as the ventral tegmental area (VTA) and laterodorsal tegmental areas (LDTg), involved with reward processing [82]. Ghrelin binds to neurons in VTA, where increases dopamine neuronal activity and dopamine turnover in the nucleus accumbens [83]. The neurotransmitter dopamine is of primary importance for incentive motivation [3]. Furthermore, ghrelin signaling at the level of the VTA appears to be important for feeding effects as intra-VTA injection of ghrelin increases the intake of palatable food [84].

## 3. CRF Components and the Regulation of Energy Homeostasis

The mammalian CRF family comprises several peptides such as CRF, urocortin (Ucn), UcnII, and UcnIII [85]. In addition to integrate endocrine and autonomic and behavioral responses to stress via activation of the hypothalamic-pituitary-adrenal (HPA) axis [86, 87], considerable evidence suggests a role for the CRF system in the regulation of energy balance [88]. In recent years, vast new endogenous functions have been attributed to this family, including regulation of food intake and satiety, gastrointestinal motility, vascular tone and development, hearing, and cardiac function, demonstrating the ubiquitous importance of the CRF family [89]. 

The CRF system is involved in energy homeostasis via direct central actions independent of HPA axis control [90], with dysfunctions of CRF system hypothesized to participate in the pathophysiology of obesity and eating disorders [7, 10, 91]. Most pharmacological data concerning the regulation of energy balance by the CRF system have been obtained during acute administration of CRF and Ucns which dose-dependently inhibits food intake [5]. However, whereas central administration of CRF has an effect on feeding behavior that is rather short (1–6 h) in duration, central administration of Ucn has an effect that persists 12 or 24 hours [92]. In addition, urocortin was found to have the most potent and prolonged inhibitory effect on decreasing food intake among the CRF family peptides, when administered peripherally [93, 94]. Also Ucn II and Ucn III play an important role in regulating food intake [94, 95].

Furthermore, the feeding stimulatory effect of NPY is attenuated by administration of CRF [88]. Different studies suggests that CRF is a hypothalamic regulatory factor that inhibits feeding induced by NPY [88, 96]. I.c.v. pretreatment with a CRF receptor antagonist, *α*-helical CRF [9–41], potentiated feeding induced by NPY, suggesting that brain CRF systems attenuate intake under conditions of evoked appetite [88, 96].

In addition to energy intake, there is evidence that CRF and Ucn also affect energy expenditure. Chronic administration of CRF and Ucn dose-dependently decreases body weight, with a great impact of CRF [94]. Acute administration of CRF activates the sympathetic nervous system (SNS), brown fat tissue (BAT) thermogenesis [5, 97], elevates norepinephrine release in several brain areas [98, 99], increased uncoupling protein-1 in BAT [100], increases body temperature [101], and reduces carcass fat [6]. In this regard, central infusion of CRF induces a state of negative energy balance that is partially a function of its effects on food intake and partially a function of its activating effects on the sympathetic nervous system. In contrast, UCN appears to induce a less dramatic state of negative energy balance that is primarily dependent on changes in food intake and does not appear to involve sympathetic nervous system activation [102]. Consistent with this idea, brown adipose fat pad weights and adrenal tissue weights increased in CRF-treated rats. Furthermore, chronic central administration of CRF resulted in elevated corticosterone, cholesterol, triglycerides, and free fatty acids (FFA), all of those suggesting SNS-induced lipolysis. When a selective antagonist of the CRFR2 receptor, antisauvagine-30 (ASV-30), was administrated, it could clearly attenuated the effects of both UCN and CRF on food intake, but it did not affect the SNS or HPA variables that were altered by chronic CRF-infusion. Interestingly, ASV-30 alone also increased food intake and glucose levels, providing the evidence that antagonism of endogenous CRF can result in increased feeding [102]. The effects of the urocortin on the microstructure of ingestive behavior are analogous to those of the dexfenfluramine, a serotoninergic agonist which suppresses appetite. Anorexic and thermogenic properties of serotonin receptor agonist have been shown to be dependent on brain [88]. 

The distribution and regulation of both known subtypes of CRF receptor, CRFR1, and CRFR2 further support the relevance of brain CRF systems in energy balance regulation. In particular, CRFR1 receptor are broadly distributed in brain with high densities observed in cortical and limbic regions relevant for regulation of hypophysiotropic secretion and sympathetic outflow mediated by brainstem autonomic nuclei, and the CRF2 receptors are focally distributed with high densities in subcortical regions such as the olfactory bulb, lateral septum, and ventromedial hypothalamus. Moreover, levels of protein and message for CRF itself and both CRF receptors are altered by experimental conditions such as anorexic drug treatment or fasting which disturbs energy homeostasis [88].

Experiments conducted in rats have suggested that paraventricular hypothalamic nucleus is the site of the anorexic effect, but the observations that PVH lesions do not prevent the CRF-mediated anorexic effects are consistent with the view that the anoretic actions of CRF and urocortin can be exerted at extra PVH sites. The chronic infusion of urocortin into the arcuate-ventromedial region causes anorexia suggests that both the ventromedial hypothalamic nucleus, which express the CRFR2, and the arcuate nucleus, which express CRFR1, can be sites of the anorectic effects of CRF-related peptides [88].

Several lines of evidence indicate that CRF-induced anxiogenic effects, ACTH secretion, and locomotor activation are mediated by the activation of CRF receptor type 1 [103, 104]. Bradbury et al. have reported that stress-induced anorexia may involve stimulation of CRFR1 during the first hours of the response; a similar reduction in the amount of food and water consumed following the treatment with urocortin was observed in CRFR1 KO mice [105]. Moreover, a decrease in food-water intake was observed in CRFR1 KO and wt mice following i.c.v CRF treatment [103]. In addition, CRFR1 KO mice appear to exhibit normal food intake over a 24 h period relative to wild-type controls, further suggesting that receptors in brain other than CRFR1 regulate normal food intake [106]. CRFR2 may mediate the appetite-suppressing effects of CRF-like peptides [103], since the selective downregulation of CRFR2 mRNA with an antisense oligonucleotide attenuates CRF-induced anorexia. The demonstration that expression of the CRFR2 gene is reduced in obese, diabetic, and food-deprived rats and increased following i.c.v. infusions of leptin is also consistent with a role for the CRFR2 in the regulation of energy balance [88]. 

The role of CRFR2 receptor in energy balance may primarily mediate the appetite suppressing effects of CRF rather than the metabolic effects of this peptide [102]. Genetic deletion of CRF2 receptors increased food intake during the dark phase of the light/dark cycle. Microstructural analyses indicated that this orexigenic effect was due to increased meal size. CRF2 pathway endogenously reinforces the satiating value of food at the circadian time of greatest spontaneous intake. This suggests that the CRF2 pathway might be involved in the processing of gut-derived satiation signals or might potentiate their action [107, 108].

Studies conducted in the rat suggest that CRFergic activity is reduced in obesity and food-deprived rats [109]. The obese Zucker rat is more sensitive than its lean counterpart to the effects of a central infusion of CRF [85]. There is evidence that the expression of CRF-binding protein (CRF-BP) is reduced in the medial preoptic area and the basolateral complex of the amygdala in obese and food-deprived rats. In addition, obesity and food deprivation reduce the expression of CRFR2 receptor mRNA within the VMH [88]. In fact, obese rodents readily react to stressful and food deprivation which can even induce, in genetically obese animals, a neurogenic-stress-like response that strongly stimulates the CRF system. Nonetheless, there are adaptations of the CRF system in obesity (or following food deprivation). Food deprivation could concur to reduce the CRF tone [85].

However, there is increasing evidence that, in humans, abdominal obesity phenotype may be characterized by a hyperactivation or hyperresponsiveness of the HPA axis [110]. In the Cushing syndrome, hypercortisolism results in abdominal obesity [109].

### 3.1. Stress Response: Activation of the Hypothalamic-Pituitary-Adrenal Axis and Glucocorticoids (Gcs) Feedback

Stress triggers a physiological response that involves the coordinated control of multiple motor, hormonal, and vegetative systems that are engaged to reestablish homeostasis in the face of the stress perturbation, either real or perceived, of the internal and/or external environment. The neuroendocrine component of the stress response is characterized by the activation of the hypothalamic-pituitary-adrenal axis which results in high levels of glucocorticoids in the blood [6, 9, 10].

The resulting stress levels of circulating glucocorticoids give rise to multiple, complex physiological effects with highly variable kinetics throughout the whole organism, including effects on glucose metabolism and mobilization in different tissues, regulation of immune and inflammatory responses, cardiovascular effects, neuroendocrine actions, and effects on cognition [9].

A main feedback effect of glucocorticoids is to suppress the activation of the HPA axis, inhibiting HPA hormone secretion and precipitating the termination of the neuroendocrine stress response. These inhibitory feedback effects on HPA axis activation are thought to occur in the hippocampus, the hypothalamus, and the pituitary gland [9, 111].

Acutely, glucocorticoids increase utilizable energy by promoting glycogen and protein metabolism in liver and muscle, respectively, along with enhancing catecholamine-induced lipolysis in adipose tissue [112]. 

Acutely (within hours) glucocorticoids directly inhibit further activity in the hypothalamo-pituitary-adrenal axis, but the chronic actions (across days) of these steroids on brain are directly excitatory. Chronically high concentrations of Gcs act in three ways that are functionally congruent. (i) Gcs increase the expression of CRF mRNA in the central nucleus of the amygdala, a critical node in the emotional brain. CRF enables recruitment of a chronic stress-response network. (ii) Gcs increase the salience of pleasurable or compulsive activities (ingesting sucrose, fat, and drugs, or wheel-running). This motivates ingestion of “comfort food.” (iii) Gcs act systemically to increase abdominal fat depots [113].

As glucocorticoids increase, insulin secretion also increases, as it is well known from the strong association of Cushing's syndrome with type 2 diabetes. It appears that insulin plays a profound role in food selection whereas Gcs determine the motivation for selecting these foods, perhaps thought their actions on dopamine secretion in the nucleus accumbens [1].

## 4. Ghrelin and CRF System

Ghrelin and CRF have opposed functions in the control of food intake. Ghrelin is a hunger signal, released by the stomach into the circulation and produced in a subset of hypothalamic neurons [70]. Its secretion is triggered to counteract further deficit of storage and to prevent starvation. CRF causes anorexia and also activates the sympathetic nervous system in addition to its role as a major regulator of the hypothalamic-pituitary-adrenal axis [114]. 

However, increasing evidence suggests an interplay between the CRF and ghrelin systems. Ghrelin expression is present in afferents to CRF-expressing neurons [41]. In addition, peripheral and i.c.v. ghrelin injection increases CRF mRNA expression *in vivo* in the hypothalamus of rats [115] and mice [116] *in vitro* [82] “*in vivo*” and “*in vitro*”, in the hypothalamus of rats and mice [85, 112, 116] and has a potent ACTH-releasing activity [43]. Functional interactions between these systems in the control of gastrointestinal motility [116] have been demonstrated. In addition, ghrelin has anxiety-like effects [117–119].

Furthermore, the CRF system interacts with ghrelin. Following acute stress, a rise in either gastric ghrelin mRNA or total plasma ghrelin has been observed, also tail pinch stress significantly increased ghrelin mRNA expression [116, 120], although, recently, it has been demonstrated that increasing ghrelin through caloric restriction decreases anxiety and depressive-like behavior via GHS-R1a signaling [119].

Indeed, Ucn increased acylated and desacylated ghrelin levels in the gastric body and decreased their levels in plasma, and decreased preproghrelin mRNA levels in the gastric body. In addition, Ucn-induced reduction of plasma ghrelin and food intake were restored by CRFR2 but not CRFR1 and Ucn-induced reduction of food intake was restored by exogenous ghrelin [91]. 

Endogenously as well as exogenously induced hypercortisolism leads to a significant decrease in plasma ghrelin levels in humans, indicating a possible feedback mechanism between gastric ghrelin secretion and the activity levels of the HPA [121]. 

Within the brain, the expression of GHS-R1a is remarkably high in the hypothalamus-pituitary unit, in agreement with its impact on anterior pituitary function as well as with its influence in the control of appetite, food intake, and energy balance [61, 82]. But, mRNA encoding the GHS-R was also expressed in several other discrete regions of the brain (rat), such as dentate gyrus, CA2 and CA3 regions of the hippocampal formation, thalamic regions, and several nuclei within the brainstem including pars compacta of the substantia nigra, ventral tegmental area, median and dorsal raphe nuclei, Edinger-Westphal nucleus, laterodorsal nucleus, and facial nerve [82]. It looks like that a relationship between ghrelin and the CRF system exists, since ghrelin receptor has been detected in the paraventricular nucleus, the principal source of CRF, and in the Edinger-Westphal nuclei, the primary site of urocortin expression.

### 4.1. Brain Circuitry in the Hypothalamus: Connecting Ghrelin

Ghrelin activates Agrp/NPY neurons, thereby stimulating food intake. GHS receptor mRNA is expressed in 94% of arcuate neurons that express NPY [122]. The ARC-NPY/Agrp neurons project dorsally and anteriorly into the perifornical lateral hypothalamic area, paraventriculat nucleus, dorsomedial nucleus of the hypothalamus, and medial preoptic area [74]. NPY projections to the PVH are derived from the arcuate nucleus and the brainstem, and synaptic contacts between NPY terminals and CRF neurons have been demonstrated. Available pharmacological evidence suggests that NPY exerts a stimulatory effect on CRF neurons. It is possible that under normal conditions, brief activation of the ARC NPY system might not only trigger a feeding response, but also activate counteracting mechanism as the CRF system [123]. Although no convincing Y1 receptor staining was found in CRF cell bodies, Y1-positive fibers made close appositions on CRF cell bodies in the parvocellular nucleus as well as in the periventricular zone. So, NPY may be able to indirectly modulate CRF neuronal activity by acting on presynaptic Y1 receptors to modulate the secretion of neurotransmitters or neuromodulators, which in turn modulate CRF neuronal activity [123]. The group of Smith has discovered the presence of CRFR1 colocalized to NPY neurons in the ARC, and it provided a potential neuroanatomical circuit by which CRF may inhibit NPY signaling by direct modulation of NPY neurons [124]. One possible effect of ghrelin is to increase the release of NPY onto GABAergic nerve terminals, disinhibiting the CRF neuron and thus stimulating greater CRF release into the pituitary-hypophyseal portal circulation, driving increased ACTH secretion from the pituitary [41]. The CRF receptor antagonist *α*-helical CRF9-41 significantly inhibited ghrelin-induced anxiogenic effects, so this behavior effect includes a mechanism of action involving CRF [116].

## 5. Stress and Obesity: Ghrelin as a Hedonic Signal?

### 5.1. Reward System and the Control of Food Intake

Reward is often conceptualized as if it were a single psychological process or a unitary feature of a reinforcing stimulus. It is sometimes identified with the pleasure or hedonic impact of a stimulus and is viewed by some as necessarily subjective in nature. However, reward is not a unitary process, but instead a constellation of multiple processes many of which can be separately identified in behavior, especially after the component processes are dissociated by brain manipulations [125]. 

Certain foods, particularly those rich in sugars and fat, are potent rewards that promote eating [126, 127] and triggered learned associations between the stimulus and the reward (conditioning) [128]. Several neurotransmitters, including DA, as well neuropeptides involved in homeostatic regulation of food intake, are implicated in the rewarding effects of food. Dopamine is a key neurotransmitter modulating reward, which it does mainly through its projections from the ventral tegmental area (VTA) [128].

The VTA dopamine (DA) projection to nucleus accumbens (NAcc) has been implicated in the control of behaviors motivated by rewards [129–131]. The VTA also contains DA neurons that project to medial prefrontal cortex (PFC), a structure linked functionally to temporal organization of goal-directed behaviors [131]. 

The mesolimbic dopamine projections, originating from neuronal cell populations in the VTA and terminating in the ventral striatum and the prefrontal cortex, are linked to anticipatory, appetitive, or approach phases of motivated behavior and are important for anticipatory food reward and food-seeking behavior [132]. 

The extensive glutamatergic afferents to DA neurons from regions involved with sensory (insula or primary gustatory cortex), homeostatic (hypothalamus), reward (NAc), emotional (amygdala and hippocampus), and multimodal (orbifrontal cortex (OFC) for salience attribution) modulate their activity in response to rewards and to conditioned cues [128, 133]. Specifically, projections from the amygdala and the OFC to DA neurons and NAc are involved in conditioned responses to food [128, 134].

### 5.2. Ghrelin as a Hedonic Signal?

Chronic stress induces changes in mood, feeding, and metabolism by a poorly understood neurobiological mechanism. Elevated stress hormones and palatable food intake and the consequent accretion of fat can serve as feedback signals that reduce perceived stress, thus reinforcing stress-induced feeding behavior [1].

Stress not only increases glucocorticoid levels through the cellular actions of CRF in the hypothalamic-pituitary-adrenal axis, but also induces the release of CRF in extrahypothalamic brain regions, such as VTA [135, 136], where stimulates CRFR1 on VTA dopamine neurons and activates a protein-kinase-C- (PKC-) dependent enhancement of Ih, which led to increased cell firing [137]. This activation may be implicated in the interaction between stress, dopamine, and motivation which is important for many behaviors and psychiatric disorders such as depression [138–140] drug abuse [136, 141], and schizophrenia [138, 142]. 

Ghrelin has recently emerged as one of the major contributing factors to reward-driven feeding that can override the state of satiation [3, 81, 84, 143]. The ghrelin receptor, GHS-R1A, is also expressed in tegmental and mesolimbic areas involved in reward, such as the VTA and laterodorsal tegmental areas (LDTg). Intracerebroventricular injection of ghrelin has been shown to stimulate food intake [144], especially the intake of palatable food [84]. Moreover, the effects of peripheral ghrelin on food intake were blunted by intra-VTA administration of a GHS-R1A antagonist [83]. Consistent with this, peripheral treatment with a GHS-R1A antagonist decreased preference for palatable food, suppressed the ability of sweet treats to condition a place preference [84], and suppressed motivated behavior for rewarding foods, both sweet [3, 84, 143] and high-fat foods [81]. Collectively these data support the idea that the physiological role of ghrelin is to increase the incentive motivation for natural rewards such as food.

The central ghrelin signaling system emerges as a novel and interesting therapeutic target as studies in rodents have shown that ghrelin antagonists suppress the mesoaccumbal dopamine system, suppress the intake of (and preference for) palatable food, suppress the ability of rewarding foods to condition a place preference, and decrease operant responding for rewarding foods [3]. The CRF system is directly implicated in the regulation of energy balance [5, 6] and may participate in the pathophysiology of obesity and eating disorders. Additionally, increasing evidence suggests an interplay between the CRF and ghrelin systems. Further studies should determine whether ghrelin plays a role in the feeding behavior associated with stress.

## References

[1] Dallman MF (2010). Stress-induced obesity and the emotional nervous system. *Trends in Endocrinology and Metabolism*.

[2] Muccioli G, Tschöp M, Papotti M, Deghenghi R, Heiman M, Ghigo E (2002). Neuroendocrine and peripheral activities of ghrelin: implications in metabolism and obesity. *European Journal of Pharmacology*.

[3] Egecioglu E, Skibicka KP, Hansson C (2011). Hedonic and incentive signals for body weight control. *Reviews in Endocrine and Metabolic Disorders*.

[4] Skibicka KP, Hansson C, Alvarez-Crespo M, Friberg PA, Dickson SL (2011). Ghrelin directly targets the ventral tegmental area to increase food motivation. *Neuroscience*.

[5] Arase K, York DA, Shimizu H, Shargill N, Bray GA (1988). Effects of corticotropin-releasing factor on food intake and brown adipose tissue thermogenesis in rats. *American Journal of Physiology Endocrinology and Metabolism*.

[6] Rivest S, Deshaies Y, Richard D (1989). Effects of corticotropin-releasing factor on energy balance in rats are sex dependent. *American Journal of Physiology, Regulatory, Integrative and Comparative Physiology*.

[7] Connan F, Lightman SL, Landau S, Wheeler M, Treasure J, Campbell IC (2007). An investigation of hypothalamic-pituitary-adrenal axis hyperactivity in anorexia nervosa: the role of CRH and AVP. *Journal of Psychiatric Research*.

[8] Doyon C, Moraru A, Richard D (2004). The corticotropin-releasing factor system as a potential target for antiobesity drugs. *Drug News and Perspectives*.

[9] Tasker JB (2006). Rapid glucocorticoid actions in the hypothalamus as a mechanism of homeostatic integration. *Obesity*.

[10] Lemmens SG, Rutters F, Born JM, Westerterp-Plantenga MS (2011). Stress augments food 'wanting' and energy intake in visceral overweight subjects in the absence of hunger. *Physiology and Behavior*.

[11] Dieguez C, Casanueva FF (2000). Ghrelin: a step forward in the understanding of somatotroph cell function and growth regulation. *European Journal of Endocrinology*.

[12] Howard AD, Feighner SD, Cully DF (1996). A receptor in pituitary and hypothalamus that functions in growth hormone release. *Science*.

[13] Kojima M, Hosoda H, Date Y, Nakazato M, Matsuo H, Kangawa K (1999). Ghrelin is a growth-hormone-releasing acylated peptide from stomach. *Nature*.

[14] Banks WA, Tschöp M, Robinson SM, Heiman ML (2002). Extent and direction of ghrelin transport across the blood-brain barrier is determined by its unique primary structure. *Journal of Pharmacology and Experimental Therapeutics*.

[15] Matsumoto M, Hosoda H, Kitajima Y (2001). Structure-activity relationship of ghrelin: pharmacological study of ghrelin peptides. *Biochemical and Biophysical Research Communications*.

[16] Gutierrez JA, Solenberg PJ, Perkins DR (2008). Ghrelin octanoylation mediated by an orphan lipid transferase. *Proceedings of the National Academy of Sciences of the United States of America*.

[17] Yang J, Brown MS, Liang G, Grishin NV, Goldstein JL (2008). Identification of the acyltransferase that octanoylates ghrelin, an appetite-stimulating peptide hormone. *Cell*.

[18] Gualillo O, Lago F, Dieguez C (2008). Introducing GOAT: a target for obesity and anti-diabetic drugs?. *Trends in Pharmacological Sciences*.

[19] Lim CT, Kola B, Korbonits M (2011). The ghrelin/GOAT/GHS-R system and energy metabolism. *Reviews in Endocrine and Metabolic Disorders*.

[20] Lim CT, Kola B, Grossman A, Korbonits M (2011). The expression of ghrelin O-acyltransferase (GOAT) in human tissues. *Endocrine Journal*.

[21] Kirchner H, Gutierrez JA, Solenberg PJ (2009). GOAT links dietary lipids with the endocrine control of energy balance. *Nature Medicine*.

[22] Broglio F, Benso A, Gottero C (2003). Non-acylated ghrelin does not possess the pituitaric and pancreatic endocrine activity of acylated ghrelin in humans. *Journal of Endocrinological Investigation*.

[23] Ariyasu H, Takaya K, Tagami T (2001). Stomach is a major source of circulating ghrelin, and feeding state determines plasma ghrelin-like immunoreactivity levels in humans. *The The Journal of Clinical Endocrinology and Metabolism*.

[24] Date Y, Kojima M, Hosoda H (2000). Ghrelin, a novel growth hormone-releasing acylated peptide, is synthesized in a distinct endocrine cell type in the gastrointestinal tracts of rats and humans. *Endocrinology*.

[25] Dornonville de la Cour C, Björkqvist M, Sandvik AK (2001). A-like cells in the rat stomach contain ghrelin and do not operate under gastrin control. *Regulatory Peptides*.

[26] Lee HM, Wang G, Englander EW, Kojima M, Greeley GH (2002). Ghrelin, a new gastrointestinal endocrine peptide that stimulates insulin secretion: enteric distribution, ontogeny, influence of endocrine, and dietary manipulations. *Endocrinology*.

[27] Sakata I, Nakamura K, Yamazaki M (2002). Ghrelin-producing cells exist as two types of cells, closed- and opened-type cells, in the rat gastrointestinal tract. *Peptides*.

[28] Gnanapavan S, Kola B, Bustin SA (2002). The tissue distribution of the mRNA of ghrelin and subtypes of its receptor, GHS-R, in humans. *The Journal of Clinical Endocrinology and Metabolism*.

[29] Hosoda H, Kojima M, Matsuo H, Kangawa K (2000). Ghrelin and des-acyl ghrelin: two major forms of rat ghrelin peptide in gastrointestinal tissue. *Biochemical and Biophysical Research Communications*.

[30] Date Y, Nakazato M, Hashiguchi S (2002). Ghrelin is present in pancreatic *α*-cells of humans and rats and stimulates insulin secretion. *Diabetes*.

[31] Caminos JE, Gualillo O, Lago F (2005). The endogenous growth hormone secretagogue (Ghrelin) is synthesized and secreted by chondrocytes. *Endocrinology*.

[32] Mori K, Yoshimoto A, Takaya K (2000). Kidney produces a novel acylated peptide, ghrelin. *FEBS Letters*.

[33] Gualillo O, Caminos JE, Blanco M (2001). Ghrelin, a novel placental-derived hormone. *Endocrinology*.

[34] Tena-Sempere M, Barreiro ML, González LC (2002). Novel expression and functional role of ghrelin in rat testis. *Endocrinology*.

[35] Caminos JE, Tena-Sempere M, Gaytán F (2003). Expression of ghrelin in the cyclic and pregnant rat ovary. *Endocrinology*.

[36] Gaytan F, Barreiro ML, Chopin LK (2003). Immunolocalization of ghrelin and its functional receptor, the type 1a growth hormone secretagogue receptor, in the cyclic human ovary. *The Journal of Clinical Endocrinology and Metabolism*.

[37] Andreis PG, Malendowicz LK, Trejter M (2003). Ghrelin and growth hormone secretagogue receptor are expressed in the rat adrenal cortex: evidence that ghrelin stimulates the growth, but not the secretory activity of adrenal cells. *FEBS Letters*.

[38] Korbonits M, Grossman AB (2004). Ghrelin: update on a novel hormonal system. *European Journal of Endocrinology*.

[39] Caminos JE, Nogueiras R, Blanco M (2003). Cellular distribution and regulation of ghrelin messenger ribonucleic acid in the rat pituitary gland. *Endocrinology*.

[40] Korbonits M, Kojima M, Kangawa K, Grossman A (2001). Presence of ghrelin in normal and adenomatous human pituitary. *Endocrine*.

[41] Cowley MA, Smith RG, Diano S (2003). The distribution and mechanism of action of ghrelin in the CNS demonstrates a novel hypothalamic circuit regulating energy homeostasis. *Neuron*.

[42] Kamegai J, Tamura H, Shimizu T, Ishii S, Sugihara H, Wakabayashi I (2000). Central effect of ghrelin, an endogenous growth hormone secretagogue, on hypothalamic peptide gene expression. *Endocrinology*.

[43] Arvat E, Maccario M, di Vito L (2001). Endocrine activities of ghrelin, a natural growth hormone secretagogue (GHS), in humans: comparison and interactions with hexarelin, a nonnatural peptidyl GHS, and GH-releasing hormone. *The Journal of Clinical Endocrinology and Metabolism*.

[44] Druce MR, Wren AM, Park AJ (2005). Ghrelin increases food intake in obese as well as lean subjects. *International Journal of Obesity*.

[45] Tang-Christensen M, Vrang N, Ortmann S, Bidlingmaier M, Horvath TL, Tschöp M (2004). Central administration of ghrelin and agouti-related protein (83-132) increases food intake and decreases spontaneous locomotor activity in rats. *Endocrinology*.

[46] Wren AM, Small CJ, Ward HL (2000). The novel hypothalamic peptide ghrelin stimulates food intake and growth hormone secretion. *Endocrinology*.

[47] Wren AM, Small CJ, Abbott CR (2001). Ghrelin causes hyperphagia and obesity in rats. *Diabetes*.

[48] Nakazato M, Murakami N, Date Y (2001). A role for ghrelin in the central regulation of feeding. *Nature*.

[49] Kamegai J, Tamura H, Shimizu T, Ishii S, Sugihara H, Wakabayashi I (2001). Chronic central infusion of ghrelin increases hypothalamic neuropeptide Y and agouti-related protein mRNA levels and body weight in rats. *Diabetes*.

[50] Shintani M, Ogawa Y, Ebihara K (2001). Ghrelin, an endogenous growth hormone secretagogue, is a novel orexigenic peptide that antagonizes leptin action through the activation of hypothalamic neuropeptide Y/Y1 receptor pathway. *Diabetes*.

[51] Tschop M, Smiley DL, Heiman ML (2000). Ghrelin induces adiposity in rodents. *Nature*.

[52] Rüter J, Kobelt P, Tebbe JJ (2003). Intraperitoneal injection of ghrelin induces Fos expression in the paraventricular nucleus of the hypothalamus in rats. *Brain Research*.

[53] Lawrence CB, Snape AC, Baudoin FMH, Luckman SM (2002). Acute central ghrelin and GH secretagogues induce feeding and activate brain appetite centers. *Endocrinology*.

[54] Chen HY, Trumbauer ME, Chen AS (2004). Orexigenic action of peripheral ghrelin is mediated by neuropeptide Y and agouti-related protein. *Endocrinology*.

[55] Asakawa A, Inui A, Kaga T (2001). Ghrelin is an appetite-stimulatory signal from stomach with structural resemblance to motilin. *Gastroenterology*.

[56] Seoane LM, López M, Tovar S, Casanueva FF, Señarís R, Diéguez C (2003). Agouti-related peptide, neuropeptide Y, and somatostatin-producing neurons are targets for ghrelin actions in the rat hypothalamus. *Endocrinology*.

[57] Cummings DE, Purnell JQ, Frayo RS, Schmidova K, Wisse BE, Weigle DS (2001). A preprandial rise in plasma ghrelin levels suggests a role in meal initiation in humans. *Diabetes*.

[58] Bagnasco M, Kalra PS, Kalra SP (2002). Ghrelin and leptin pulse discharge in fed and fasted rats. *Endocrinology*.

[59] Tolle V, Bassant MH, Zizzari P (2002). Ultradian rhythmicity of ghrelin secretion in relation with gh, feeding behavior, and sleep-wake patterns in rats. *Endocrinology*.

[60] Inui A (2001). Ghrelin: an orexigenic and somatotrophic signal from the stomach. *Nature Reviews Neuroscience*.

[61] Giordano R, Picu A, Broglio F (2004). Ghrelin, hypothalamus-pituitary-adrenal (HPA) axis and Cushing's syndrome. *Pituitary*.

[62] Tschöp M, Weyer C, Tataranni PA, Devanarayan V, Ravussin E, Heiman ML (2001). Circulating ghrelin levels are decreased in human obesity. *Diabetes*.

[63] Otto B, Cuntz U, Fruehauf E (2001). Weight gain decreases elevated plasma ghrelin concentrations of patients with anorexia nervosa. *European Journal of Endocrinology*.

[64] de Vriese C, Perret J, Delporte C (2010). Focus on the short- and long-term effects of ghrelin on energy homeostasis. *Nutrition*.

[65] Fetissov SO, Hamze Sinno M, Coquerel Q (2008). Emerging role of autoantibodies against appetite-regulating neuropeptides in eating disorders. *Nutrition*.

[66] Fetissov SO, Hamze Sinno M, Coëffier M (2008). Autoantibodies against appetite-regulating peptide hormones and neuropeptides: putative modulation by gut microflora. *Nutrition*.

[67] Terashi M, Asakawa A, Harada T (2011). Ghrelin reactive autoantibodies in restrictive anorexia nervosa. *Nutrition*.

[68] Goldstone AP, Thomas EL, Brynes AE (2004). Elevated fasting plasma ghrelin in Prader-Willi syndrome adults is not solely explained by their reduced visceral adiposity and insulin resistance. *The Journal of Clinical Endocrinology and Metabolism*.

[69] English PJ, Ghatei MA, Malik IA, Bloom SR, Wilding JPH (2002). Food fails to suppress ghrelin levels in obese humans. *The Journal of Clinical Endocrinology and Metabolism*.

[70] Horvath TL, Diano S, Sotonyi P, Heiman M, Tschöp M (2001). Minireview: ghrelin and the regulation of energy balance-a hypothalamic perspective. *Endocrinology*.

[71] Shiiya T, Nakazato M, Mizuta M (2002). Plasma ghrelin levels in lean and obese humans and the effect of glucose on ghrelin secretion. *The Journal of Clinical Endocrinology and Metabolism*.

[72] Toshinai K, Mondal MS, Nakazato M (2001). Upregulation of ghrelin expression in the stomach upon fasting, insulin-induced hypoglycemia, and leptin administration. *Biochemical and Biophysical Research Communications*.

[73] Briggs DI, Enriori PJ, Lemus MB, Cowley MA, Andrews ZB (2010). Diet-induced obesity causes ghrelin resistance in arcuate NPY/AgRP neurons. *Endocrinology*.

[74] Williams G, Bing C, Cai XJ, Harrold JA, King PJ, Liu XH (2001). The hypothalamus and the control of energy homeostasis: different circuits, different purposes. *Physiology and Behavior*.

[75] Baskin DG, Breininger JF, Schwartz MW (1999). Leptin receptor mrna identifies a subpopulation of neuropeptide Y neurons activated by fasting in rat hypothalamus. *Diabetes*.

[76] Broberger C, Johansen J, Johansson C, Schalling M, Hökfelt T (1998). The neuropeptide Y/agouti gene-related protein (AGRP) brain circuitry in normal, anorectic, and monosodium glutamate-treated mice. *Proceedings of the National Academy of Sciences of the United States of America*.

[77] Wang L, Saint-Pierre DH, Taché Y (2002). Peripheral ghrelin selectively increases Fos expression in neuropeptide Y—synthesizing neurons in mouse hypothalamic arcuate nucleus. *Neuroscience Letters*.

[78] Dallman MF (2003). Filling the interstices: ghrelin neurons plug several holes in regulation of energy balance. *Neuron*.

[79] Gualillo O, Lago F, Gómez-Reino J, Casanueva FF, Dieguez C (2003). Ghrelin, a widespread hormone: insights into molecular and cellular regulation of its expression and mechanism of action. *FEBS Letters*.

[80] Wren AM, Small CJ, Fribbens CV (2002). The hypothalamic mechanisms of the hypophysiotropic action of ghrelin. *Neuroendocrinology*.

[81] Perello M, Sakata I, Birnbaum S (2010). Ghrelin increases the rewarding value of high-fat diet in an orexin-dependent manner. *Biological Psychiatry*.

[82] Guan XM, Yu H, Palyha OC (1997). Distribution of mRNA encoding the growth hormone secretagogue receptor in brain and peripheral tissues. *Molecular Brain Research*.

[83] Abizaid A, Liu ZW, Andrews ZB (2006). Ghrelin modulates the activity and synaptic input organization of midbrain dopamine neurons while promoting appetite. *Journal of Clinical Investigation*.

[84] Egecioglu E, Jerlhag E, Salomé N (2010). Ghrelin increases intake of rewarding food in rodents. *Addiction Biology*.

[85] Richard D, Lin Q, Timofeeva E (2002). The corticotropin-releasing factor family of peptides and CRF receptors: their roles in the regulation of energy balance. *European Journal of Pharmacology*.

[86] Heinrichs SC, Koob GF (2004). Corticotropin-releasing factor in brain: a role in activation, arousal, and affect regulation. *The Journal of Pharmacology and Experimental Therapeutics*.

[87] Koob GF, Heinrichs SC (1999). A role for corticotropin releasing factor and urocortin in behavioral responses to stressors. *Brain Research*.

[88] Heinrichs SC, Richard D (1999). The role of corticotropin-releasing factor and urocortin in the modulation of ingestive behavior. *Neuropeptides*.

[89] Bale TL, Vale WW (2004). CRF and CRF Receptors: role in Stress Responsivity and Other Behaviors. *Annual Review of Pharmacology and Toxicology*.

[90] Cone RD (2000). The corticotropin-releasing hormone system and feeding behavior-a complex web begins to unravel. *Endocrinology*.

[91] Yakabi K, Noguchi M, Ohno S (2011). Urocortin 1 reduces food intake and ghrelin secretion via CRF2 receptors. *American Journal of Physiology—Endocrinology and Metabolism*.

[92] Contarino A, Gold LH (2002). Targeted mutations of the corticotropin-releasing factor system: effects on physiology and behavior. *Neuropeptides*.

[93] Spina M, Merlo-Pich E, Chan RKW (1996). Appetite-suppressing effects of urocortin, a CRF-related neuropeptide. *Science*.

[94] Tanaka C, Asakawa A, Ushikai M (2009). Comparison of the anorexigenic activity of CRF family peptides. *Biochemical and Biophysical Research Communications*.

[95] Chen P, Vaughan J, Donaldson C, Vale W, Li C (2010). Injection of Urocortin 3 into the ventromedial hypothalamus modulates feeding, blood glucose levels, and hypothalamic POMC gene expression but not the HPA axis. *American Journal of Physiology—Endocrinology and Metabolism*.

[96] Heinrichs SC, Menzaghi F, Pich EM, Hauger RL, Koob GF (1993). Corticotropin-releasing factor in the paraventricular nucleus modulates feeding induced by neuropeptide Y. *Brain Research*.

[97] Brown MR, Fisher LA, Spiess J, Rivier C, Rivier J, Vale W (1982). Corticotropin-releasing factor: actions on the sympathetic nervous system and metabolism. *Endocrinology*.

[98] Dunn AJ, Berridge CW (1987). Corticotropin-releasing factor administration elicits a stress-like activation of cerebral catecholaminergic systems. *Pharmacology Biochemistry and Behavior*.

[99] Emoto H, Tanaka M, Koga C, Yokoo H, Tsuda A, Yoshida M (1993). Corticotropin-releasing factor activates the noradrenergic neuron system in the rat brain. *Pharmacology Biochemistry and Behavior*.

[100] Kotz CM, Wang C, Levine AS, Billington CJ (2002). Urocortin in the hypothalamic PVN increases leptin and affects uncoupling proteins-1 and -3 in rats. *American Journal of Physiology—Regulatory Integrative and Comparative Physiology*.

[101] Rothwell NJ (1990). Central effects of CRF on metabolism and energy balance. *Neuroscience and Biobehavioral Reviews*.

[102] Cullen MJ, Ling N, Foster AC, Pelleymounter MA (2001). Urocortin, corticotropin releasing factor-2 receptors and energy balance. *Endocrinology*.

[103] Contarino A, Dellu F, Koob GF (2000). Dissociation of locomotor activation and suppression of food intake induced by CRF in CRFR1-deficient mice. *Endocrinology*.

[104] Timpl P, Spanagel R, Sillaber I (1998). Impaired stress response and reduced anxiety in mice lacking a functional corticotropin-releasing hormone receptor 1. *Nature Genetics*.

[105] Bradbury MJ, Mcburnie MI, Denton DA, Lee KF, Vale WW (2000). Modulation of urocortin-induced hypophagia and weight loss by corticotropin-releasing factor receptor 1 deficiency in mice. *Endocrinology*.

[106] Smith GW, Aubry JM, Dellu F (1998). Corticotropin releasing factor receptor 1-deficient mice display decreased anxiety, impaired stress response, and aberrant neuroendocrine development. *Neuron*.

[107] Tabarin A, Diz-Chaves Y, Consoli D (2007). Role of the corticotropin-releasing factor receptor type 2 in the control of food intake in mice: a meal pattern analysis. *European Journal of Neuroscience*.

[108] Woods SC (2004). Gastrointestinal satiety signals I. An overview of gastrointestinal signals that influence food intake. *American Journal of Physiology—Gastrointestinal and Liver Physiology*.

[109] Mastorakos G, Zapanti E (2004). The hypothalamic-pituitary-adrenal axis in the neuroendocrine regulation of food intake and obesity: the role of corticotropin releasing hormone. *Nutritional Neuroscience*.

[110] Vicennati V, Ceroni L, Gagliardi L, Gambineri A, Pasquali R (2002). Response of the hypothalamic pituitary-adrenocortical axis to high-protein/fat and high-carbohydrate meals in women with different obesity phenotypes. *The Journal of Clinical Endocrinology and Metabolism*.

[111] Keller-Wood ME, Dallman MF (1984). Corticosteroid inhibition of ACTH secretion. *Endocrine Reviews*.

[112] Brindley DN (1995). Role of glucocorticoids and fatty acids in the impairment of lipid metabolism in the metabolic syndrome. *International Journal of Obesity and Related Metabolic Disorders*.

[113] Dallman MF, Pecoraro N, Akana SF (2003). Chronic stress and obesity: a new view of "comfort food". *Proceedings of the National Academy of Sciences of the United States of America*.

[114] Schwartz MW, Woods SC, Porte D, Seeley RJ, Baskin DG (2000). Central nervous system control of food intake. *Nature*.

[115] Johnstone LE, Srisawat R, Kumarnsit E, Leng G (2005). Hypothalamic expression of NPY mRNA, vasopressin mRNA and CRF mRNA in response to food restriction and central administration of the orexigenic peptide GHRP-6. *Stress*.

[116] Asakawa A, Inui A, Kaga T (2001). A role of ghrelin in neuroendocrine and behavioral responses to stress in mice. *Neuroendocrinology*.

[117] Carlini VP, Varas MM, Cragnolini AB, Schiöth HB, Scimonelli TN, De Barioglio SR (2004). Differential role of the hippocampus, amygdala, and dorsal raphe nucleus in regulating feeding, memory, and anxiety-like behavioral responses to ghrelin. *Biochemical and Biophysical Research Communications*.

[118] Hansson C, Haage D, Taube M, Egecioglu E, Salomé N, Dickson SL (2011). Central administration of ghrelin alters emotional responses in rats: behavioural, electrophysiological and molecular evidence. *Neuroscience*.

[119] Lutter M, Sakata I, Osborne-Lawrence S (2008). The orexigenic hormone ghrelin defends against depressive symptoms of chronic stress. *Nature Neuroscience*.

[120] Kristenssson E, Sundqvist M, Astin M (2006). Acute psychological stress raises plasma ghrelin in the rat. *Regulatory Peptides*.

[121] Otto B, Tschöp M, Heldwein W, Pfeiffer AF, Diederich S (2004). Endogenous and exogenous glucocorticoids decrease plasma ghrelin in humans. *European Journal of Endocrinology*.

[122] Tannenbaum GS, Lapointe M, Beaudet A, Howard AD (1998). Expression of growth hormone secretagogue-receptors by growth hormone-releasing Hormone neurons in the mediobasal hypothalamus. *Endocrinology*.

[123] Li C, Chen P, Smith MS (2000). Corticotropin releasing hormone neurons in the paraventricular nucleus are direct targets for neuropeptide Y neurons in the arcuate nucleus: an anterograde tracing study. *Brain Research*.

[124] Campbell RE, Grove KL, Smith MS (2003). Distribution of corticotropin releasing hormone receptor immunoreactivity in the rat hypothalamus: coexpression in neuropeptide Y and dopamine neurons in the arcuate nucleus. *Brain Research*.

[125] Berridge KC, Robinson TE (1998). What is the role of dopamine in reward: hedonic impact, reward learning, or incentive salience?. *Brain Research Reviews*.

[126] de Araujo I, Ren X, Ferreira JG (2010). Metabolic sensing in brain dopamine systems. *Results and Problems in Cell Differentiation*.

[127] Lenoir M, Serre F, Cantin L, Ahmed SH (2007). Intense sweetness surpasses cocaine reward. *PLoS ONE*.

[128] Volkow ND, Wang GJ, Baler RD (2011). Reward, dopamine and the control of food intake: implications for obesity. *Trends in Cognitive Sciences*.

[129] di Chiara G, Imperato A (1988). Drugs abused by humans preferentially increase synaptic dopamine concentrations in the mesolimbic system of freely moving rats. *Proceedings of the National Academy of Sciences of the United States of America*.

[130] Koob GF (1992). Neural mechanisms of drug reinforcement. *Annals of the New York Academy of Sciences*.

[131] Richardson NR, Gratton A (1998). Changes in medial prefrontal cortical dopamine levels associated with response-contingent food reward: an electrochemical study in rat. *Journal of Neuroscience*.

[132] Bassareo V, di Chiara G (1999). Modulation of feeding-induced activation of mesolimbic dopamine transmission by appetitive stimuli and its relation to motivational state. *European Journal of Neuroscience*.

[133] Geisler S, Wise RA (2008). Functional implications of glutamatergic projections to the ventral tegmental area. *Reviews in the Neurosciences*.

[134] Petrovich GD (2011). Forebrain circuits and control of feeding by learned cues. *Neurobiology of Learning and Memory*.

[135] Hauger RL, Risbrough V, Brauns O, Dautzenberg FM (2006). Corticotropin releasing factor (CFR) receptor signaling in the central nervous system: new molecular targets. *CNS and Neurological Disorders Drug Targets*.

[136] Wang B, Shaham Y, Zitzman D, Azari S, Wise RA, You ZB (2005). Cocaine experience establishes control of midbrain glutamate and dopamine by corticotropin-releasing factor: a role in stress-induced relapse to drug seeking. *Journal of Neuroscience*.

[137] Wanat MJ, Hopf FW, Stuber GD, Phillips PEM, Bonci A (2008). Corticotropin-releasing factor increases mouse ventral tegmental area dopamine neuron firing through a protein kinase C-dependent enhancement of Ih. *The Journal of Physiology*.

[138] Banki CM, Bissette G, Arato M, O'Connor L, Nemeroff CB (1987). CSF corticotropin-releasing factor-like immunoreactivity in depression and schizophrenia. *American Journal of Psychiatry*.

[139] Gershon AA, Vishne T, Grunhaus L (2007). Dopamine D2-like receptors and the antidepressant response. *Biological Psychiatry*.

[140] Valdez GR (2006). Development of CRF1 receptor antagonists as antidepressants and anxiolytics: progress to date. *CNS Drugs*.

[141] McFarland K, Davidge SB, Lapish CC, Kalivas PW (2004). Limbic and motor circuitry underlying footshock-induced reinstatement of cocaine-seeking behavior. *Journal of Neuroscience*.

[142] Beninger RJ (2006). Dopamine and incentive learning: a framework for considering antipsychotic medication effects. *Neurotoxicity Research*.

[143] Skibicka KP, Hansson C, Egecioglu E, Dickson SL Role of ghrelin in food reward: impact of ghrelin on sucrose self-administration and mesolimbic dopamine and acetylcholine receptor gene expression.

[144] Naleid AM, Grace MK, Cummings DE, Levine AS (2005). Ghrelin induces feeding in the mesolimbic reward pathway between the ventral tegmental area and the nucleus accumbens. *Peptides*.

